# Supplementary material for: Hot spot prediction in protein-protein interactions by an ensemble system
Source: BMC Syst Biol. 2018 Dec 31;12(Suppl 9):132. doi: 10.1186/s12918-018-0665-8 (PMC6311905; doi:10.1186/s12918-018-0665-8)
Supplement: Supplementary file 2 — All relevant MATLAB code. A simple Matlab implement of our predictor. (ZIP 19,742 kb) [file 12918_2018_665_MOESM2_ESM.zip › Additional file 2/Read ASA/ASA website/SKEMPI/2QJAC.htm]

 NetSurfP - Protein Surface Accessibility and Secondary Structure Predictions

  

|  |  |  |
| --- | --- | --- |
|  |  | NetSurfP - Protein Surface Accessibility and Secondary Structure Predictions  Technical University of Denmark |

  


---

```
# For publication of results, please cite:
# A generic method for assignment of reliability scores applied to solvent accessibility predictions.
# Bent Petersen, Thomas Nordahl Petersen, Pernille Andersen, Morten Nielsen and Claus Lundegaard
# BMC Structural Biology 2009, 9:51 doi:10.1186/1472-6807-9-51
#
# Column 1: Class assignment - B for buried or E for Exposed - Threshold: 25% exposure, but not based on RSA
# Column 2: Amino acid
# Column 3: Sequence name
# Column 4: Amino acid number
# Column 5: Relative Surface Accessibility - RSA
# Column 6: Absolute Surface Accessibility
# Column 7: Z-fit score for RSA prediction
# Column 8: Probability for Alpha-Helix
# Column 9: Probability for Beta-strand
# Column 10: Probability for Coil
E T  2QJAC                  1    0.861 119.351   0.341   0.003   0.003   0.994
E L  2QJAC                  2    0.281  51.488  -0.398   0.005   0.045   0.951
E P  2QJAC                  3    0.334  47.451  -1.218   0.004   0.085   0.910
B F  2QJAC                  4    0.257  51.500  -0.382   0.005   0.336   0.660
B L  2QJAC                  5    0.060  11.078  -0.821   0.002   0.816   0.182
E K  2QJAC                  6    0.306  62.862   1.080   0.001   0.900   0.099
B C  2QJAC                  7    0.020   2.822   0.666   0.001   0.959   0.040
B Y  2QJAC                  8    0.246  52.677   1.202   0.001   0.900   0.099
B C  2QJAC                  9    0.017   2.373   1.060   0.003   0.718   0.279
E S  2QJAC                 10    0.300  35.113   0.811   0.004   0.197   0.799
E G  2QJAC                 11    0.595  46.850  -2.355   0.019   0.141   0.840
E H  2QJAC                 12    0.510  92.805   0.201   0.020   0.205   0.775
B C  2QJAC                 13    0.052   7.343  -1.087   0.018   0.088   0.893
E P  2QJAC                 14    0.657  93.186  -0.471   0.018   0.047   0.935
E D  2QJAC                 15    0.670  96.605  -1.835   0.018   0.019   0.964
E D  2QJAC                 16    0.555  79.932  -1.828   0.018   0.019   0.964
E A  2QJAC                 17    0.481  53.061  -1.315   0.018   0.019   0.964
E I  2QJAC                 18    0.494  91.427  -0.554   0.018   0.047   0.935
E N  2QJAC                 19    0.427  62.469  -0.899   0.018   0.047   0.935
B N  2QJAC                 20    0.250  36.629  -0.539   0.018   0.088   0.893
E T  2QJAC                 21    0.288  40.015   1.003   0.021   0.451   0.528
B C  2QJAC                 22    0.036   5.054  -0.842   0.022   0.552   0.426
E I  2QJAC                 23    0.361  66.730   0.911   0.022   0.359   0.619
E T  2QJAC                 24    0.516  71.597  -0.698   0.056   0.142   0.802
E N  2QJAC                 25    0.619  90.651  -0.665   0.058   0.017   0.925
E G  2QJAC                 26    0.282  22.209  -0.950   0.053   0.043   0.903
E H  2QJAC                 27    0.296  53.879   0.839   0.074   0.484   0.442
B C  2QJAC                 28    0.033   4.619   0.047   0.087   0.683   0.230
B F  2QJAC                 29    0.076  15.333   0.852   0.085   0.775   0.140
B A  2QJAC                 30    0.146  16.045   0.675   0.085   0.775   0.140
B I  2QJAC                 31    0.118  21.811   0.661   0.072   0.855   0.073
B I  2QJAC                 32    0.099  18.241   0.352   0.011   0.918   0.071
B E  2QJAC                 33    0.274  47.815  -0.061   0.011   0.918   0.071
E E  2QJAC                 34    0.328  57.284   0.069   0.011   0.918   0.071
B D  2QJAC                 35    0.273  39.325  -0.326   0.023   0.655   0.322
E D  2QJAC                 36    0.609  87.786  -0.574   0.022   0.359   0.619
E Q  2QJAC                 37    0.718 128.253  -0.989   0.018   0.088   0.893
E G  2QJAC                 38    0.372  29.253  -0.721   0.019   0.141   0.840
E E  2QJAC                 39    0.451  78.755  -0.334   0.023   0.655   0.322
E T  2QJAC                 40    0.397  55.050   0.570   0.011   0.918   0.071
E T  2QJAC                 41    0.340  47.116   0.634   0.006   0.962   0.032
E L  2QJAC                 42    0.269  49.181   1.077   0.011   0.918   0.071
E T  2QJAC                 43    0.379  52.540  -0.330   0.018   0.846   0.136
B S  2QJAC                 44    0.227  26.581   0.180   0.004   0.616   0.381
B G  2QJAC                 45    0.251  19.722  -1.555   0.004   0.420   0.576
B C  2QJAC                 46    0.062   8.761  -1.432   0.005   0.262   0.733
B L  2QJAC                 47    0.130  23.748  -0.234   0.019   0.141   0.840
E G  2QJAC                 48    0.342  26.884   1.208   0.005   0.015   0.979
E L  2QJAC                 49    0.364  66.593  -0.009   0.018   0.019   0.964
E E  2QJAC                 50    0.699 122.168  -0.960   0.018   0.019   0.964
E G  2QJAC                 51    0.644  50.714  -2.057   0.018   0.019   0.964
E S  2QJAC                 52    0.406  47.630  -0.839   0.018   0.047   0.935
E D  2QJAC                 53    0.400  57.669   0.223   0.022   0.359   0.619
B F  2QJAC                 54    0.067  13.467  -0.722   0.022   0.552   0.426
B Q  2QJAC                 55    0.277  49.526   0.501   0.023   0.655   0.322
B C  2QJAC                 56    0.024   3.313  -0.715   0.022   0.552   0.426
E R  2QJAC                 57    0.369  84.432   0.064   0.005   0.336   0.660
E D  2QJAC                 58    0.362  52.164  -1.262   0.004   0.085   0.910
E T  2QJAC                 59    0.555  76.923  -0.541   0.018   0.088   0.893
E P  2QJAC                 60    0.471  66.821  -1.581   0.018   0.047   0.935
E I  2QJAC                 61    0.463  85.729  -0.334   0.018   0.019   0.964
E P  2QJAC                 62    0.411  58.349  -1.628   0.018   0.019   0.964
E H  2QJAC                 63    0.511  92.987  -1.305   0.018   0.019   0.964
E Q  2QJAC                 64    0.544  97.141  -0.504   0.018   0.019   0.964
E R  2QJAC                 65    0.455 104.195  -1.125   0.018   0.047   0.935
E R  2QJAC                 66    0.346  79.234  -0.051   0.020   0.205   0.775
E S  2QJAC                 67    0.287  33.590   0.419   0.023   0.655   0.322
B I  2QJAC                 68    0.094  17.353   0.861   0.018   0.846   0.136
B E  2QJAC                 69    0.253  44.217   0.609   0.018   0.846   0.136
B C  2QJAC                 70    0.028   3.889   0.358   0.023   0.655   0.322
B C  2QJAC                 71    0.060   8.410   0.986   0.021   0.451   0.528
E R  2QJAC                 72    0.457 104.699  -0.059   0.019   0.141   0.840
E T  2QJAC                 73    0.503  69.711  -0.070   0.018   0.019   0.964
E N  2QJAC                 74    0.569  83.228  -1.050   0.058   0.017   0.925
B L  2QJAC                 75    0.211  38.671   0.551   0.115   0.016   0.868
B C  2QJAC                 76    0.051   7.174  -0.391   0.115   0.016   0.868
B N  2QJAC                 77    0.266  39.001  -0.526   0.115   0.016   0.868
E Q  2QJAC                 78    0.553  98.855  -0.395   0.058   0.017   0.925
E Y  2QJAC                 79    0.442  94.562  -0.332   0.018   0.047   0.935
B L  2QJAC                 80    0.095  17.468   0.480   0.018   0.088   0.893
E Q  2QJAC                 81    0.457  81.567   0.910   0.004   0.085   0.910
B P  2QJAC                 82    0.238  33.744  -0.993   0.004   0.085   0.910
E T  2QJAC                 83    0.634  87.991  -0.698   0.005   0.045   0.951
E L  2QJAC                 84    0.286  52.348   0.629   0.005   0.015   0.979
E P  2QJAC                 85    0.782 110.923  -0.541   0.003   0.003   0.994

Explain the output.  Go back.
```

---
